# IL-22R Ligands IL-20, IL-22, and IL-24 Promote Wound Healing in Diabetic *db/db* Mice

**DOI:** 10.1371/journal.pone.0170639

**Published:** 2017-01-26

**Authors:** Ganesh Kolumam, Xiumin Wu, Wyne P. Lee, Jason A. Hackney, Jose Zavala-Solorio, Vineela Gandham, Dimitry M. Danilenko, Puneet Arora, Xiaoting Wang, Wenjun Ouyang

**Affiliations:** 1 Department of Biomedical Imaging, Genentech, South San Francisco, California, United States of America; 2 Department of Immunology, Genentech, South San Francisco, California, United States of America; 3 Department of Bioinformatics and Computational Biology, Genentech, South San Francisco, California, United States of America; 4 Department of Molecular Biology, Genentech, South San Francisco, California, United States of America; 5 Department of Safety Assessment Pathology, Genentech, South San Francisco, California, United States of America; 6 Department of Early Clinical Development, Genentech, South San Francisco, California, United States of America; University of Illinois at Chicago, UNITED STATES

## Abstract

Diabetic foot ulcers (DFU) are one of the major complications in type II diabetes patients and can result in amputation and morbidity. Although multiple approaches are used clinically to help wound closure, many patients still lack adequate treatment. Here we show that IL-20 subfamily cytokines are upregulated during normal wound healing. While there is a redundant role for each individual cytokine in this subfamily in wound healing, mice deficient in IL-22R, the common receptor chain for IL-20, IL-22, and IL-24, display a significant delay in wound healing. Furthermore, IL-20, IL-22 and IL-24 are all able to promote wound healing in type II diabetic *db/db* mice. Mechanistically, when compared to other growth factors such as VEGF and PDGF that accelerate wound healing in this model, IL-22 uniquely induced genes involved in reepithelialization, tissue remodeling and innate host defense mechanisms from wounded skin. Interestingly, IL-22 treatment showed superior efficacy compared to PDGF or VEGF in an infectious diabetic wound model. Taken together, our data suggest that IL-20 subfamily cytokines, particularly IL-20, IL-22, and IL-24, might provide therapeutic benefit for patients with DFU.

## Introduction

Normal cutaneous wound healing can be divided into at least four overlapping phases, hemostasis (or coagulation), inflammation, re-epithelialization, and remodeling [1–4]. Many different cell types, such as platelets, keratinocytes, fibroblasts, endothelial cells and macrophages, participate in wound healing processes. Significant delay of wound healing steps results in chronic wounds such as diabetic foot ulcers (DFU), pressure ulcers and venous ulcers [5, 6]. With increasing incidence of diabetes mellitus worldwide, DFU often leads to substantial morbidity and mortality and presents a significant unmet medical need [7, 8]. Five-year mortality rate for a neuropathic DFU is approximately 50%, about the same as colon cancer. Approximately 80,000 amputations annually were caused by unhealed diabetic ulcer in the United States. Although neuropathy, minor foot trauma and foot deformity are considered as the major triggers of the DFU, deficiencies in angiogenic responses, leukocyte recruitment and macrophage function, extracellular matrix deposition, epidermal barrier function, and fibroblast activities contribute to the delayed wound healing in individuals with diabetes [6, 9]. In addition, uncontrolled infections in some DFU patients can further hinder the wound healing process. Given the complexity of the wound healing progress and various defects in DFU patients, proper management of wound requires multidisciplinary approaches, such as debridement, offloading, moist wound care, surgeries to help revascularization and control of infection, and promoting healing with various growth factors [10, 11]. To date, growth factors, including epidermal growth factor (EGF) family, fibroblast growth factor (FGF) family, platelet derived growth factor (PDGF), vascular endothelial growth factor (VEGF), and transforming growth factor (TGF) family factors have been shown to facilitate wound healing process through various mechanisms [5, 12]. For example, VEGF significantly expedited the wound healing process in diabetic mouse models through promoting angiogenesis, lymphangiogenesis, and recruitment of leukocytes [13, 14]. Both FGF and PDGF are able to accelerate wound healing process [15]. In various preclinical models, PDGF demonstrated consistent functions to enhance the granulation process, while the improvement in re-epithelialization was only observed in certain models [16]. Although many of these growth factors have also been tested in clinic to treat DFU patients, only recombinant human PDGF (Becaplermin or Regranex) is approved by FDA for this indication. However, its usage is limited in clinic due to relative mild benefit, high cost and safety concerns [17]. With increasing incidence of diabetes mellitus worldwide, diabetic foot ulcer often leads to substantial morbidity and mortality and presents a significant unmet medical need [7, 8].

Proinflammatory cytokines, such as GM-CSF, IL-1, IL-6 and TNF-α also exert essential roles during wound healing [5]. Recently, IL-20 subfamily cytokines, IL-19, IL-20, IL-22 and IL-24, have been demonstrated to regulate wound healing responses both in vitro and in vivo [18]. Among these cytokines, IL-20, IL-22 and IL-24 all bind to a common receptor subunit, IL-22R [19–23]. When IL-22R pairs with IL-20Rb chain, it engages IL-20 and IL-24, while when IL-22R couples with IL10Rb chain, it only serves as a receptor for IL-22. Additionally, IL-20 and IL-24 signal through IL20Ra and IL-20Rb pair, which is also shared by IL-19 [22]. Upon binding to their receptors, IL-19, IL-20, IL-22 and IL-24, activate Stat3 signaling pathways [22]. The receptors of IL-20 subfamily cytokines are all highly expressed on human primary keratinocytes [24]. In vitro, IL-20 subfamily cytokines induce some downstream biological effects observed in psoriatic skins and in wound healing, such as hyperproliferation, differentiation, proinflammatory and antimicrobial responses, and upregulation of genes involved in tissue remodeling, from cultured human primary epidermis [24, 25]. In these studies IL-22 showed the strongest potency, followed by IL-24 and IL-20, and IL-19 being the weakest. IL-22 deficient mice display defects in wound healing, especially in the dermal compartment [26]. In this full-thickness model, lack of IL-22 led to reduced extracellular matrix production while the rate of reepithelialization was similar to that in WT mice. IL-22 directly induced the expression of extracellular matrix genes in fibroblasts. In addition, IL-22 is able to promote wound healing in a streptozotocin-induced type I diabetic model [27]. Recombinant IL-19 and IL-20 also can facilitate wound healing in a full-thickness circular wound model [28].

Based on the broad wound healing effects induced by IL-20 subfamily cytokines on primary human epidermis, we investigated whether these cytokines could provide therapeutic benefits for DFU in preclinical mouse models. We chose wound healing model in type II diabetic *db/db* mice because they exhibit significant delayed wound closure compared to other diabetic strains such as streptozocin induced diabetic mice and Akita mice [29]. Wound healing in mice is fundamentally different to that of human [30, 31]. While healing in human is predominantly driven by re-epithelialization, in murine models contraction is a major force affecting the healing process. In our study, therefore, we used a well-established splinted wound healing model to minimize the differences and restrict the contraction. We observed an induction of IL-20 subfamily cytokines IL-19, IL-20, IL-22 and IL-24 in wounded skins. The absence of the IL-22R rendered the delayed wound healing process. Fc fusion proteins of the IL-22R binding cytokines, IL-20-Fc, IL-22-Fc and IL-24-Fc can significantly stimulate the wound healing in diabetic db/db mice. Mechanistically, IL-22-Fc but not VEGF and PDGF induced unique pathways including reepithelialization, tissue remodeling and antimicrobial responses from the wound. Importantly, IL-22-Fc elicited better efficacy than VEGF and PDGF in infected diabetic wound.

## Materials and Methods

### Mice

C57BL/6 mice, 10 to 12 weeks old leptin receptor deficient mice (*db/db*; BKS.Cg-Dock7m Leprdb/+ +/J), and the lean control mice were purchased from Jackson Laboratory. The genetic deficient mice for IL-22, IL-20Rb, and IL-22R were described previously [32]. Age and gender-matched female mice were selected based on their initial blood glucose (>350 mg/dl) and were randomized into study groups. In all studies, animals with skin lesion at non-surgery sites, with alopecia or other experiments unrelated health issues were euthanized immediately and excluded from the studies. The sample size in each study is described in figure legends. There was no animal death without euthanasia during experimental intervention. During skin surgery to generate the wounds, all animals were anesthetized by inhalation of isoflurane to minimize animal suffering and distress. All animals were monitored at least once dxaily during the study. All protocols were reviewed and approved by the Genentech Institutional Animal Care and Use Committee.

### Clean wound preparation, treatment and analysis

Following induction of a surgical plane of anesthesia, the dorsal portion of the back (from the scapular area to the lumbar area) was shaved, and the stubble was removed with hair remover lotion Nair. Nair was rinsed off with clean water, and then area was cleaned with Betadine^®^ Microbicides and alcohol. The animal was placed in ventral recumbency and the area of the skin to be removed was marked using a 6-mm-diameter punch. The marked wound edge was cut off with a sterile fine scissor. A 0.5-mm thick silicone ring, 10–12 mm inside diameter, was placed around each wound. A 2-cm square of Tegaderm (3M) was placed over the wound and ring. Immediately after skin wounding, mice were intraperitoneally (i.p.) or topically applied with 50-μg IL-22-Fc, control Fc, or 20μg anti-FGFR1 (Genentech). The Fc protein treatment was done every other day until the end of the study. Wounds were monitored and wound dressings were changed every other day. The vertical and horizontal wound gaps were measured with vernier caliper. The wound gap was calculated as the average of vertical and horizontal wound gaps. The mouse IL-20-Fc, IL-22-Fc and IL-24-Fc were described previously [32].

### Infectious wound preparation, treatment and analysis

The skin area was prepared as in clean wound. Mouse was placed on its side on a sterile sheet. The dorsal skin of the chest from the midline was pulled with fingers, and the folded skin was punched through (both layers) with a 6-mm-diameter sterile biopsy punch to create two symmetrical full-thickness excisional wounds besides the midline. On day 2, wounds were inoculated locally with 100 μl sterile PBS or 1x10^6^ CFU S. aureus. Tissue glue was applied to one side of a splint and carefully placed around the wound so that the wound was centered within the splint. The glue bonds to the skin on contact. Splint was secured to the skin with four interrupted sutures of 6.0 nylon. Wounds were covered with Tegaderm sterile transparent dressing. On day 4, mice were topically applied with 30ug Fc proteins, recombinant PDGF-BB (Peprotech), or VEGFα (Genentech). The doses were selected based on previous described [13, 15]. The Fc protein treatment was done three times per week until the end of the study. Photographs of individual wounds were taken with a digital camera while animals were under anesthesia. The images were analyzed with Image J software (NIH).

### Blood glucose measurement

Blood glucose was measured by Onetouch Ultra glucometer (LifeScan Inc.).

### Luciferase reporter assay

3T3 cells overexpressing human or mouse receptor complex of IL-22R/IL-20Rb, IL-20Rs/IL-20Rb were generated. 100,000 cells were seeded in 400 μL Dulbecco's modified Eagle's medium (DMEM) medium (10% fetal bovine serum [FBS], 10 mM Hepes, 10 mM GlutaMAX, and 10 mM Pen/Strepe) in 24-well plates (Corning Inc.). Cells were then transfected with plasmids encoding the Stat3 firefly luciferase reporter construct and a control Renilla luciferase construct (Genentech) in 100 μL Opti-MEM^®^ reduced serum medium (Thermo Fisher Scientific). Next day, cells were stimulated with recombinant cytokines (R&D Systems) in serial diluted concentrations. Supernatant was discarded after 20 hours and the cells were lysed and analyzed with Dual-Luciferase^®^ Reporter 1000 Assay System (Promega). The ratio of firefly to Renilla luminescent represents the Stat3 activity. Stat3 reporter plasmid was constructed to express the firefly luciferase reporter gene under the control of a minimal CMV promoter and tandem repeats of the Sis-inducible transcriptional response element.

### RNA isolation and gene expression analysis

C57BL/6 mice were i.p. injected with 100ug IL-22-Fc or control Fc. 24 hours later, the skin of the dorsal area was rapidly shaved with razor removed and snap-frozen in Trizol (Life technologies). The tissue was homogenized with 5-mm metal beads with TissueLyser(Qiagen). mRNA was isolated with PureLink RNA kit (Life technologies). RNA was directly analyzed by One Step RT-PCR (Qiagen) on ABI7500 (Applied Biosystems). Results were normalized to those of the control Rpl19 (encoding ribosomal protein L19) and are reported as 1,000×2ΔCT. Primer/probe sets for S100a9 and Defb1 were purchased from Life technologies. For microarray analysis, wounds of *db/db* mice were topically treated with 50 ug control Fc, IL-22-Fc, PDGF-BB, or VEGFα on day 0, 2, 4, and 6. On day 7, the skin surrounded the wound was cut off as a 2-mm circle and immediately snap-frozen in RNAlater (Life technologies). Gene expression analysis was performed using an Affymetrix platform (Mouse430V2) following the manufacturer’s protocols (Affymetrix). The microarray data are available from the GEO repository under accession number GSE80419.

Raw microarray data were processed using the R programming language (http://www.r-project.org) and packages from the Bioconductor project (http://www.bioconductor.org). Probe set intensities were normalized using the RMA method [33]. Probe sets were further filtered to select a single probe set per mouse Entrez gene, choosing the probe set that showed the highest variance [34]. Statistical analysis of differential expression was performed using the limma Bioconductor package. All p-values were adjusted for multiple testing using the Benjamini-Hochberg method [35]. Genes selected for heatmap were determined by ANOVA, with at least 2-fold change across the groups, at an adjusted p-value of 0.001. Genes specific to each treatment were identified by comparing each treatment to the control and both other treatments, selecting genes that showed at least 1.5 fold higher expression in the treatment of interest than the other conditions, at an adjusted p-value of 0.05.

Gene ontology (GO) analysis was performed using the GOstats Bioconductor package [36]. GO biological process terms were tested for enrichment in each of the sets of differentially expressed genes using a conditional test of over representation, using the transcriptome represented on the microarray as the background set. GO categories were considered significant at a nominal (un-adjusted) p-value of 0.01. Functional annotation networks were created by taking genes found in over-represented GO categories as nodes, with edges drawn between genes when they were found in the same GO category. GO categories with more than 2000 genes were excluded from this analysis, as they were generally insufficiently specific to allow for detailed characterization of the genes. Functional annotation of gene clusters in networks was done by hand, with clusters being annotated by the major biological role played by genes in the cluster. Networks were visualized using Cytoscape software [37].

### Histologic evaluation

Immunohistochemistry (IHC) was performed on formalin-fixed paraffin embedded skin tissues. IHC for angiogenesis was performed using rat anti-CD31 (Clone MEC13.3, 553370, BD Biosciences) with fluorescein isothiocyanate labeled anti-rat IgG2a. The mouse skin was photographed under 5x microscope. Evaluation of routine histologic sections of wounds was done on 10% formalin-fixed, paraffin embedded and hematoxylin and eosin stained wounds harvested following complete wound closure. Representative histologic images of cross sections from completely closed wounds from each mouse were taken at a magnification of 100x. The quantification of the epidermal area in completely healed wounds was done in ImageJ

### Statistical analysis

Statistical analysis except for microarray was done with two-tailed unpaired Student’s t-test or 2- Way ANOVA. P value less than 0.05 was considered as statistically significant.

## Results

### Essential role of IL-22R in splinted wound healing model

**Fig 1:**
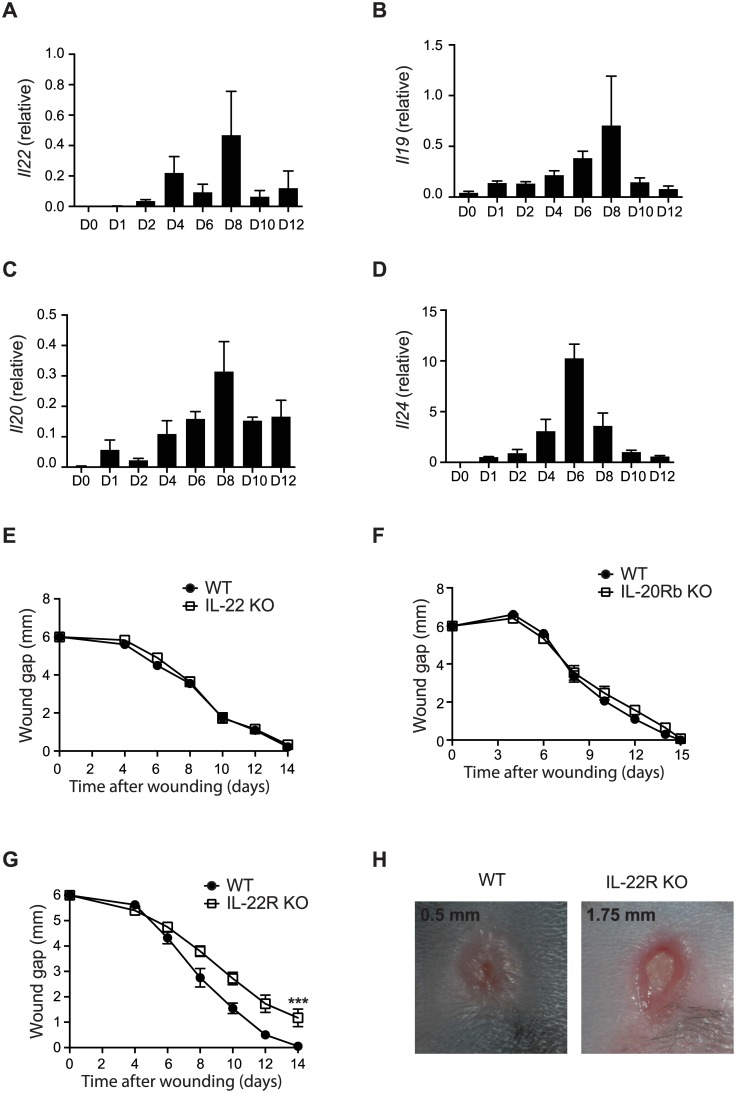
IL-22R ligands are involved in wound healing. (A-D) mRNA expression for (A) *Il22*, (B) *Il19*, (C) *Il20* and (D) *Il24* in wounded skins from C57BL/6 mice (n = 3) at different time points. (E), Wound gaps of a clean wound on the back of IL-22 KO mice (n = 9) and the littermate WT (n = 8) control mice. (F) Wound gaps of a clean wound on the back of IL-20Rb KO (n = 10) and the littermate WT (n = 9) control mice. (G) Wound gaps of a clean wound on the back of IL-22 R KO and the littermate WT (n = 10) control mice. (H). Representative pictures of a clean wound at day 12 from (G). Error bars, s.e.m. *** *P* < 0.001. 2-way ANOVA (G). Data shown are representative of two (A-F) and three (G-H) independent experiments.

To evaluate the role of IL-20 subfamily cytokines in wound healing, we first examined their expression during the wound healing process in a splinted 6-mm-diameter full thickness wound model [30]. In mice, the splinted wound is normally completely closed in about two weeks [31]. Quantitative RT-PCR analyses with mRNA samples isolated from the wounded skins at different time points revealed that all IL-20 subfamily cytokines were upregulated during the sterile wound healing process (Fig 1A–1D). IL-19, IL-20 and IL-24 were all upregulated on day 1, while IL-22 was upregulated on day 2. These data are consistent with reported upregulation of IL-22 during the normal wound healing process [26]. The expression of these cytokines peaked between day 6 and day 8. To evaluate the role of IL-20 subfamily in this splinted wound model, we tested mice deficient in either IL-22 or IL-20Rb, the common receptor chain for IL-19, IL-20, and IL-24. Consistent with previous report [26], the time course of wound closure in IL-22 deficient mice was the same as that in WT mice (Fig 1E), suggesting that in this model IL-22 had only a minimal or redundant role on reepithelialization. Similarly, the wound healing process was also normal in IL-20Rb deficient mice (Fig 1F), excluding a dominant role for other IL-20 subfamily cytokines during wound healing. These data suggest that there is a redundant role of IL-20 subfamily cytokines in cutaneous wound repair.

IL-22R is a common receptor for IL-20, IL-22 and IL-24. We then evaluated the role of IL-22R in the splinted wound healing model with IL-22R deficient mice. In contrast to the results from IL-22 deficient mice, IL-22R deficient mice showed a significant delay in wound closure in comparison to that of WT control mice (Fig 1G). This delay in wound closure first became apparent on day 8, and by day 12, the average WT wound diameter was about 0.5 mm in comparison to an average of 1.75 mm wound diameter in IL-22R deficient mice (Fig 1H). While all WT mice had complete wound closure by day 14, 70% of IL-22R deficient mice still had open wounds. These data support an essential function for IL-22R signaling during the normal wound healing process.

**Fig 2:**
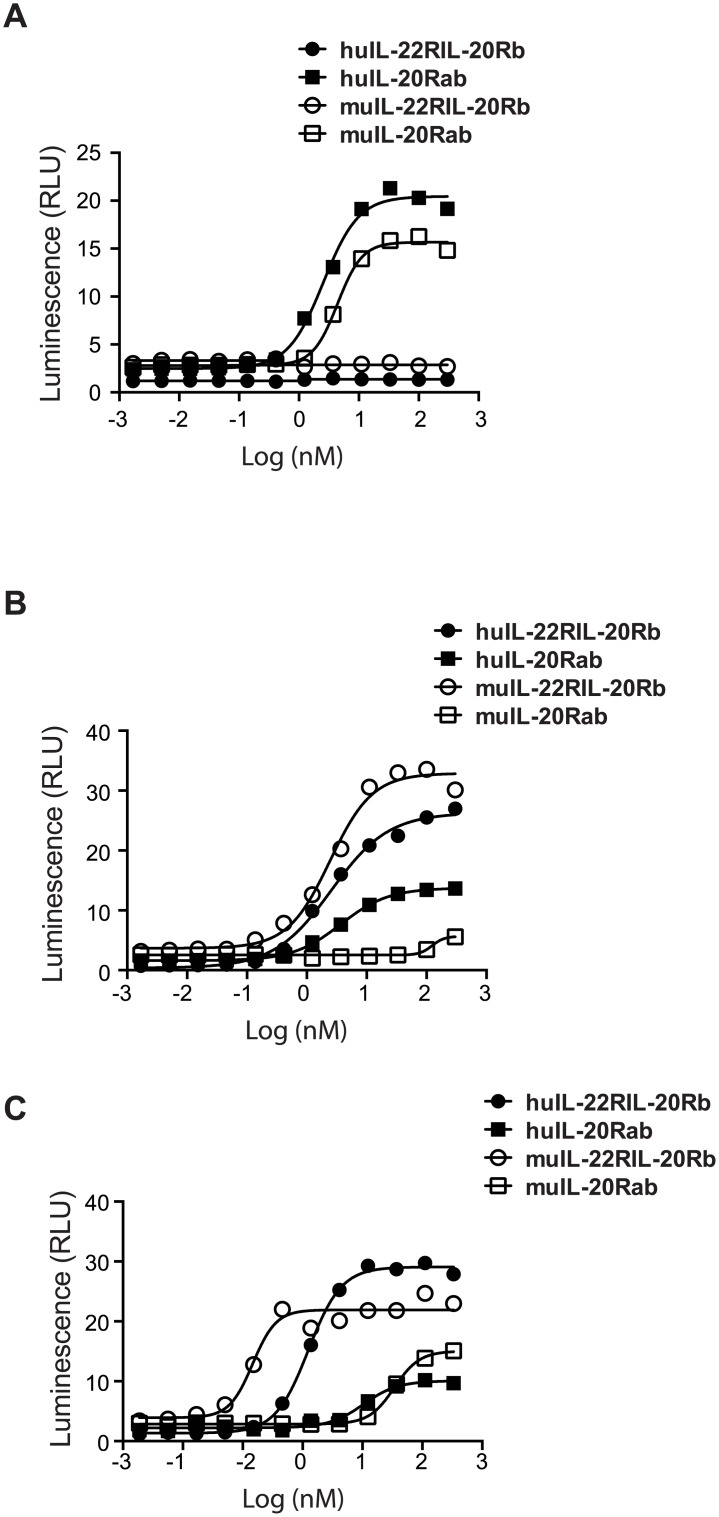
Expression of IL22 R is important for Stat3 activation by murine IL-20 and IL-24. Activities of recombinant cytokines were tested in Stat3 luciferase reporter assay on cell lines overexpressing receptors. (A) IL-19. (B) IL-20. (C) IL-24. Data shown are representative of five independent experiments.

Previous studies in human cells suggested that IL-20 and IL-24 use both IL-20Ra/IL-20Rb and IL-22R/IL-20Rb receptor pairs for their functions. However, this has not been critically examined in the murine cells. We, therefore, first examined the receptor usage by murine IL-20 subfamily cytokines in a Stat3 promoter-driven luciferase reporter assay with 3T3 cells overexpressed both murine and human receptor pairs. First, murine IL-20 subfamily cytokines were able to cross-react with human receptors (Fig 2). Murine IL-19 (Fig 2A) and IL-22 (data not shown) could induce Stat3 activity in cells expressing their designated receptor pairs similar to those reported in human system [20, 22, 23]. However, mouse IL-20 and IL-24 showed much higher potency in promoting Stat3 activity in cells bearing mouse IL-22R/IL-20Rb than that in cells expressing mouse IL-20Ra/IL-20Rb receptor pairs (Fig 2B and 2C). These data implied that in mouse IL-22R was not only essential for the function of mouse IL-22 but was also dominantly utilized by mouse IL-20 and IL-24, providing an explanation on the major role of IL-22R, but not its ligands, in wound healing.

### IL-22-Fc promotes wound healing in diabetic mouse model

**Fig 3:**
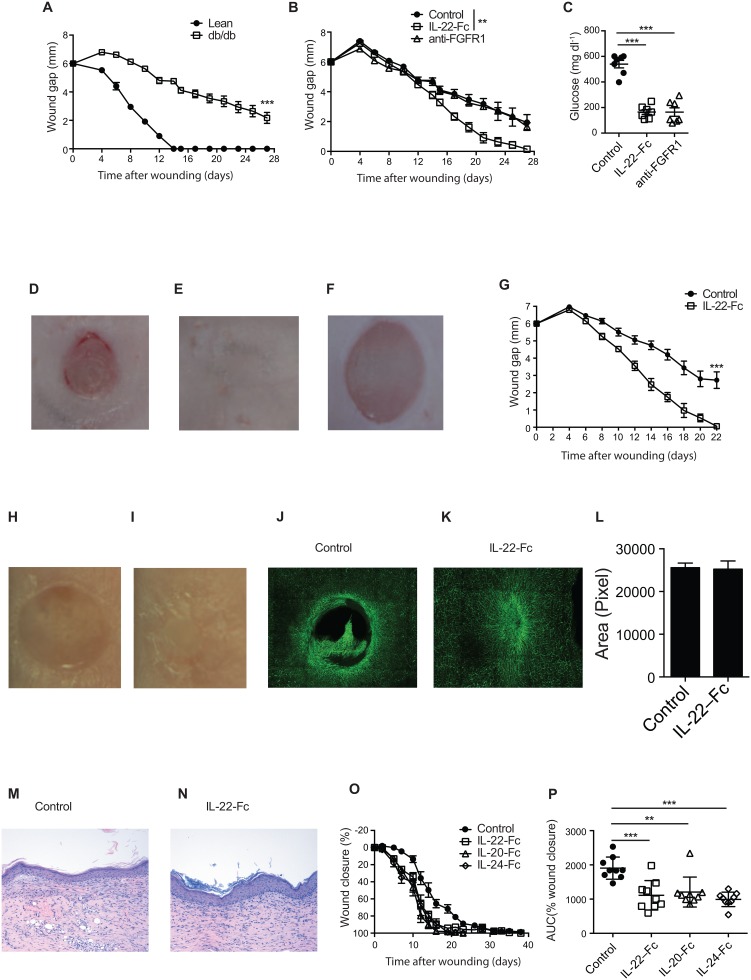
IL-22-Fc promotes wound healing in *db/db* mice. (A) Wound gaps of a clean wound on the back of *db/db* and lean control mice (n = 7). (B-F) *db/db* mice with wounds i.p. injected with control Fc, IL-22-Fc or anti-FGFR1 (n = 7). (B) Wound gaps. (C-E) Representative wound images of mice treated with (C) Control, (D) IL-22-Fc and (E) anti-FGFR1 on day 27. (F) Glucose. (G) Gaps of the wounds topically applied with control or IL-22-Fc (n = 7). (H-N) Wounds topically treated with Fc proteins. (H-I) Representative wound images on day 22 treated with (H) control and (I) IL-22-Fc. (J-K) FITC labeled anti-CD31 staining of the wounded skins on day 18 after treatment with control (J) or IL-22-Fc (K). (L) Quantification of the epidermal areas from H&E stained histologic sections from completely healed wounds from mice treated with control and IL-22Fc. (M-N) H& E stained histologic sections of healed wounds treated with control (M) or IL-22-Fc (N). (O) Wound closure (n = 6–10). (P) AUC of wound closure as in O. Error bars, s.e.m. ** *P* < 0.01, ***P < 0.001. 2-way ANOVA (A, B, G), Student’s t-test (F, P). Data shown are representative of two independent experiments.

Next, we asked whether providing exogenous IL-22R cytokine ligands could also promote the healing process in an impaired wound healing model. Diabetic *db/db* mice are used as a model to mimic the wound healing defects in DFU [29]. Splinted wounds created on *db/db* mice take more than a month to heal, while all wounds in WT can close within two weeks (Fig 3A) [29]. To evaluate the effects of IL-22R ligands on wound healing, we first systemically administrated IL-22-Fc, a fusion protein with a prolonged half-life *in vivo* [32, 38], to wounded *db/db* mice and monitored the progression of wound size. Interestingly, systemic IL-22-Fc administration significantly accelerated wound healing in these *db/db* mice. By day 27, all wounds in the IL-22-Fc treated mice were healed; whereas the wounds in control protein treated *db/db* mice remained open (Fig 3B–3D). Systemic treatment with IL-22-Fc was also able to alleviate many of the metabolic syndrome defects, including lowering serum glucose, in *db/db* mice [32]. It is therefore possible that the enhanced wound healing by IL-22-Fc could be an indirect effect via amelioration of the metabolic syndromes. To test this, we treated mice with anti-FGFR1, an antibody that provides similar metabolic benefits as those by IL-22-Fc [39]. In contrast to IL-22-Fc, anti-FGFR1 antibody failed to stimulate the wound healing process in *db/db* mice despite lowering serum glucose, suggesting that improvement of metabolic disorders alone is not sufficient to promote wound healing in this model (Fig 3B–3F).

To further demonstrate a direct effect of IL-22-Fc on promotion of cutaneous wound healing, we applied IL-22-Fc topically on the wounds. Similar to the results in mice treated with systemic IL-22-Fc, topical IL-22-Fc treatment also promoted wound healing in *db/db* mice (Figs 3G and S1). By day 22, all wounds treated topically with IL-22-Fc were completely closed, while control protein treated wounds were still open (Fig 3H and 3I). IL-22 has been shown to stimulate local VEGF production and promote angiogenesis [24, 40]. Indeed, after 18 days, IL-22-Fc treated wounds showed markedly greater vascularization as demonstrated by anti-CD31 staining compared to that of control protein treated wounds (Fig 3J and 3K). Importantly, the thickness of the epidermis and the histologic appearance of both the epidermis and dermis from completely healed wounds in both control group and IL-22-Fc treated group were very similar (Fig 3L–3N), indicating that, IL-22-Fc accelerated wound healing without altering the normal skin structure. Finally, we examined whether IL-20-Fc and IL-24-Fc had the similar functions in promoting wound healing in *db/db* mice. Similar to IL-22-Fc, IL-20-Fc and IL-24-Fc also accelerated wound closure in this model (Fig 3O and 3P). In summary, these data support the premise that the IL-22R cytokine ligands, IL-20, IL-22, and IL-24 could provide therapeutic benefit to patients with DFU.

### IL-22-Fc promotes reepitheliazation, tissue remodeling and antimicrobial responses

**Fig 4:**
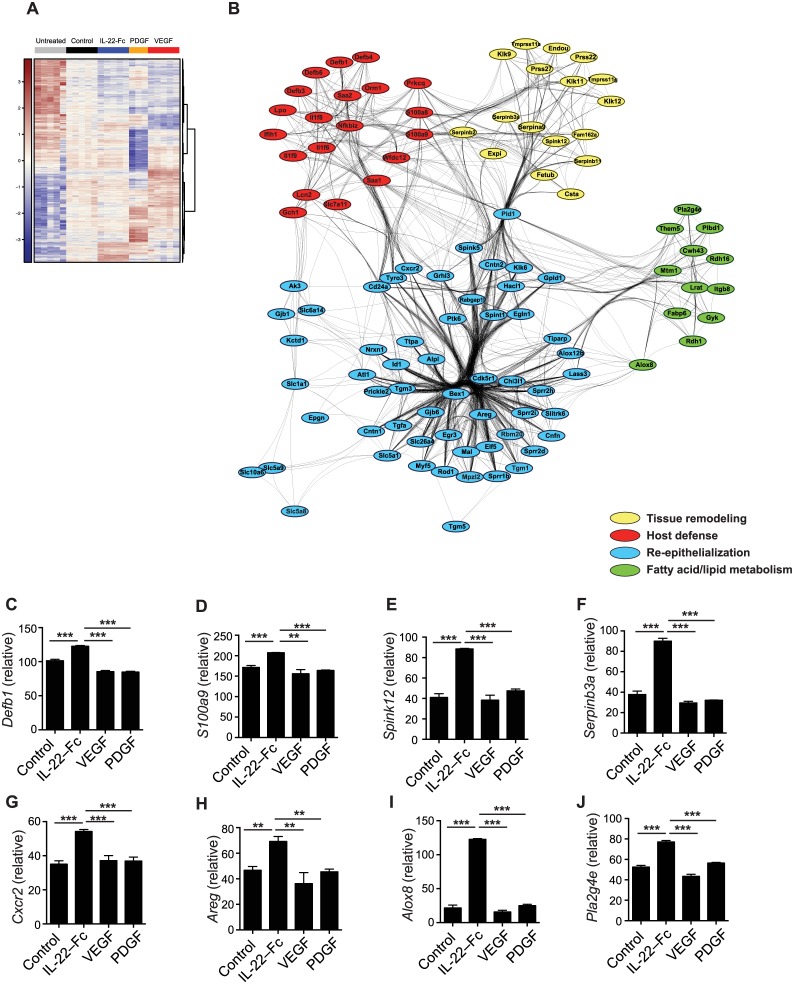
IL-22-Fc induced genes involved in wound healing. Skins were harvested on day 7 after treatment with control Fc, IL-22-Fc, PDGF or VEGF (n = 3–5). Microarray analysis was done with purified skin RNA. (A) Heat map of genes that were differentially regulated with treatments. (B) GO enrichment analysis of IL-22-Fc-specific gene expression, yielding several enriched GO categories. Genes from these categories are plotted as nodes in the network diagram, with edges indicating shared membership in an enriched GO category. (C-J) Gene expression. (C) *Defb1*, (D) *S100a9*, (E) *Spink12*, (F) *Serpinb3a*, (G) *Cxcr2*, (H) *Areg*, (I) *Alox8*, (J) *Pla2g4e*. Error bars, s.e.m. ** *P* < 0.01, ***P < 0.001. Student’s t-test (C-J).

**Fig 5:**
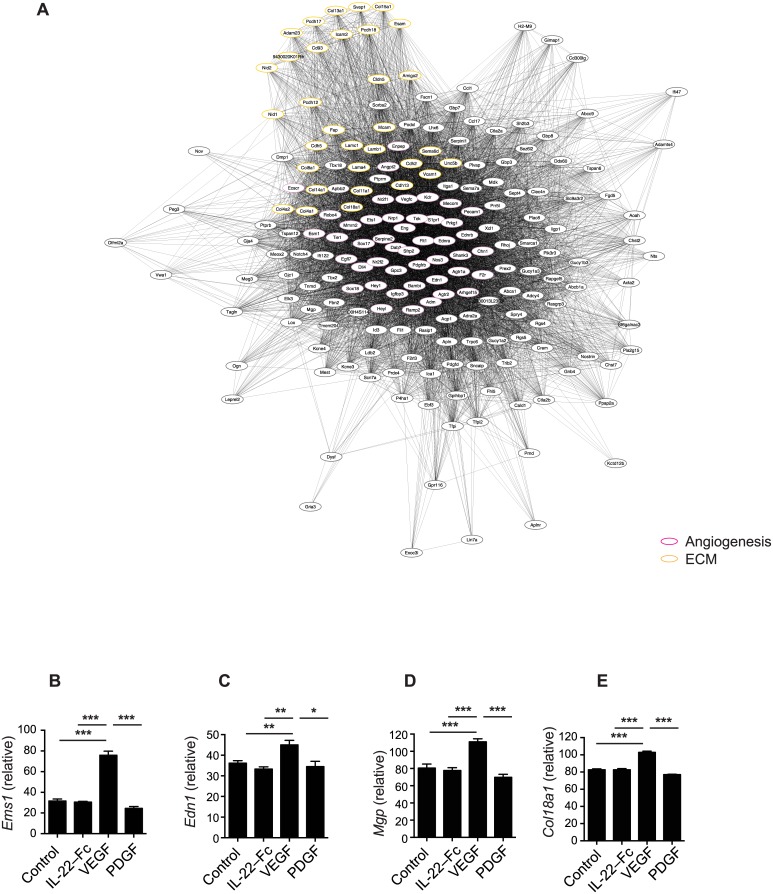
Genes up-regulated by VEGF in microarray analysis as in Fig 4. (A) GO enrichment analysis of VEGF-specific gene expression. Clusters of genes in the network diagram were manually functionally annotated according to the primary biological role played by genes in the cluster. (B-E) Gene expression of individual genes. (B) *Ems1*, (C) *Edn1*, (D) *Mgp*, (E) *Col18a1*. Error bars, s.e.m. * *P* <0.05, ** *P* < 0.01, *** *P* < 0.001. Unpaired Student’s t-test (B-E).

**Fig 6:**
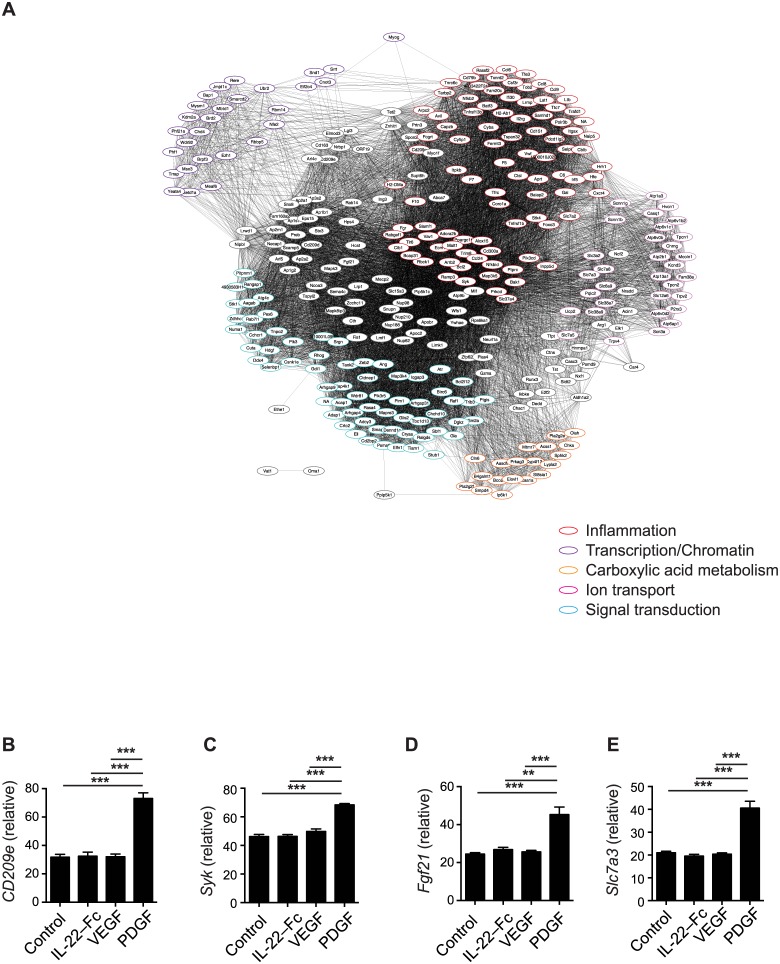
Genes up-regulated by PDGF in microarray analysis as in Fig 4. (A) GO enrichment analysis of PDGF-specific gene expression. Clusters of genes in the network diagram were manually functionally annotated according to the primary biological role played by genes in the cluster. (B-E) gene expression of individual genes. (B) *CD209e*, (C) *Syk*, (D) *Fgf21*, (E) *Slc7a3*. Error bars, s.e.m. ** *P* < 0.01, *** *P* < 0.001. Unpaired Student’s t-test (B-E).

To better understand the mechanism of IL-22-Fc in promoting wound healing we analyzed the gene expression profile of wounded skin 7 days after treatment with IL-22-Fc, and other growth factors such as PDGF and VEGF that also accelerate wound healing in the *db/db* model [13–16]. Hierarchical cluster with genes most differentially expressed based on ANOVA analysis indicated that these factors induced markedly different biological processes (Fig 4A). IL-22-Fc treatment specifically regulated more than 231 genes, 195 upregulated and 36 downregulated, while VEGF and PDGF uniquely controlled the expression of 597 genes (303 up- and 294 down-regulated) and 2051 genes (667 up- and 1384 down-regulated), respectively. Next, we functionally characterized genes uniquely upregulated by IL-22-Fc (Fig 4B), VEGF (Fig 5A), and PDGF (Fig 6A) using Gene Ontology analysis. The major pathways induced by these three cytokines were very distinct from one another. The dominant pathways promoted by IL-22-Fc included reepithelialization, tissue remodeling, host defense, and fatty acid/lipid metabolism. Fig 4C to 4J showed representative genes in each pathway that was uniquely and significantly up-regulated by IL-22-Fc treatment. Interestingly, although both PDGF and VEGF facilitated wound healing in *db/db* mice, they stimulated different downstream effectors. PDGF enhanced transcriptional/chromatin regulation, carboxylic acid metabolism, ion transport pathway, protein modification, and inflammation (Fig 6A–6E) while the downstream biology of VEGF was less diverse and was mainly focused on angiogenesis pathways and extra-cellular matrix modulation (Fig 5A–5E). These data support the conclusion that IL-22-Fc treatment provides a distinct mechanism via stimulating essential wound healing pathways including host defense (Fig 4C and 4D), tissue remodeling (Fig 4E and 4F), reepithelialization (Fig 4G and 4H), and lipid metabolism (Fig 4I and 4J) pathways that were not prominently induced by either PDGF or VEGF. Many of the genes induced by IL-22-Fc from the wound, such as *Cxcr2*, *GrhI3* and *Klk8*, are well known to participate in wound healing [41–43]. Therefore, IL-22 promotes cutaneous wound healing process through regulating multiple pathways that accelerate the skin healing. In addition, IL-22-Fc induced a strong antimicrobial response as demonstrated by upregulation of several families of antimicrobial peptides, including β-defensin, S100, Lcn2, and SAA. In addition, IL-1 family cytokines such as IL-1F6, F8, and F9 were also augmented. Interestingly, some of these antimicrobial genes were actually down regulated by PDGF and VEGF. These data suggest that IL-22-Fc treatment also is able to control microbial infections during the wound healing process.

### IL-22-Fc elicits superior wound-healing response in infected diabetic wounds

**Fig 7:**
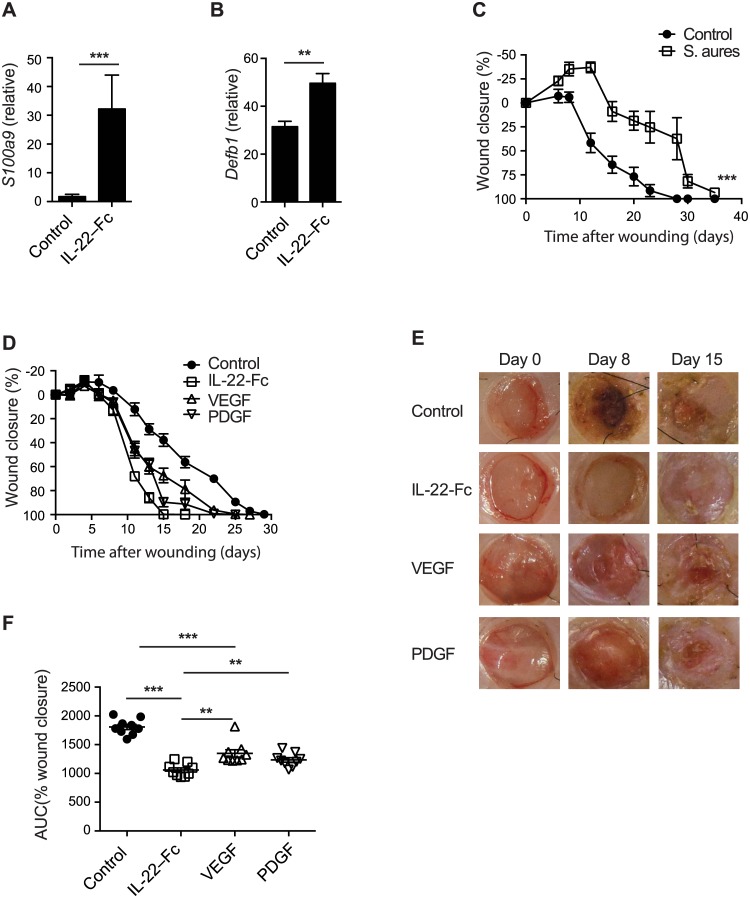
IL-22-Fc treatment is superior to VEGF and PDGF in S. aureus infected diabetic wounds. (A-B) C57BL/6 mice were i.p. injected with control or IL-22-Fc (n = 6), shaved skins on the back were harvested in 24 hours. mRNA expression of *s100a9* (A), and *Defb1* (B). (C) Wound closure of wounds infected with control (n = 8) or with S.aureus (n = 9). (D-F) *S*. *aureus* infected wounds were topically applied with control, IL-22-Fc, VEGF, or PDGF (n = 9). (D) Wound closure. (E) Representative wound images. (F) AUC of the wound closure. Error bars, s.e.m. ** *P* < 0.01, ***P < 0.001. Unpaired Student’s t-test (A, B, F). 2-way ANOVA (C). Data shown are representative of two independent experiments.

Infection is a major complication in patients with DFU, and can further hinder the healing process [8, 9]. To evaluate the potential benefit of IL-22-Fc in controlling pathogenic microbial invasion during wound repair, we first confirmed that IL-22-Fc upregulated S100A9 and β-defensin in skin samples by RT-PCR (Fig 7A and 7B). Next, we applied *Staphylococcus aureus*, a frequent bacterium identified in wounds from DFU patients [44], on the wounds of *db/db* mice. *S*. *aureus* infection further impeded the healing process in *db/db* mice (Fig 7C), mimicking the situation for infected DFU. We next tested the wound-healing promoting effects of IL-22-Fc, VEGF and PDGF in this infectious diabetic wound model. Consistent with previous reports [13, 16], both VEGF and PDGF were able to accelerate wound healing in this model (Fig 7D). IL-22-Fc, however, demonstrated an even greater acceleration of promoting wound healing when compared to these two growth factors. On day 15, nearly all IL-22-Fc treated wounds were completely closed, whereas the majority of wounds in control protein, VEGF and PDGF treated groups were still open (Fig 7D and 7E). The total wound-healing process as measured as area under the curve (AUC) was significantly improved in wounds from the IL-22-Fc treated group compared to wounds in the VEGF, PDGF or control treated groups (Fig 7F). In conclusion, these data supported the surmise that IL-22 accelerates topically wound healing process through up-regulating several key pathways and IL-22 may be able to provide benefit even in infected DFU.

## Discussion

DFU is a common complication of diabetes mellitus and is a major unmet medical need with no truly effective treatment options [7, 45]. Despite numerous clinical trials with growth factors, thus far only PDGF (Beclafermin/Regranex) has been approved for the treatment of DFU. PDGF has some benefit in achieving complete healing, but this clinical benefit is minimal in comparison with other standard cares which resulted in very narrow usage of this drug in clinic practice. In this study, we provided two lines of experimental data to support an important role of IL-20 subfamily cytokines in the wound healing process. First, the IL-20 subfamily cytokines are upregulated and indispensible during the normal wound healing responses. Second, IL-20, IL-22, and IL-24 can accelerate epithelial closure in a diabetic wound healing model. By evaluating multiple different genetically deficient mice, we found that each individual cytokine appeared to have a redundant role in driving epithelial closure following wounding, as mice deficient with either IL-22 or the common receptor chain IL-20Rb had a normal time course for wound healing. These results are consistent with previous findings in IL-22 knockout mice in wound healing model [26]. However, as demonstrated by delayed wound closure observed in IL-22R deficient mice, the IL-20 subfamily as a whole plays a critical role in wound healing. Most importantly, we demonstrated that several IL-20 subfamily cytokines, including IL-22, IL-20 and IL-24, were able to promote wound healing in diabetic *db/db* mice.

Although IL-20 and IL-24 are known to signal through two different receptor pairs, we demonstrate here that IL-22R paired with IL-20Rb is the dominant receptor used by these two cytokines in the murine system. As a result, IL-22R deficient mice not only have no IL-22 signaling, but are also deficient in both the IL-20 and IL-24 signaling pathways. Even though the function of IL-19 remains intact, previous studies have shown that IL-19 is the weakest cytokine within IL-20 subfamily as far as triggering downstream responses in epidermal keratinocytes [24]. Thus, IL-22R deficiency results in diminishing the majority of the biological functions of the IL-20 subfamily of cytokines, which provides a possible explanation of why mice with an IL-22R deficiency but not IL-22 and IL-20Rb deficiencies exhibit a delay in wound closure.

Importantly, our study demonstrated that IL-20 subfamily cytokines especially IL-22, may be able to facilitate wound healing process through various mechanisms, including reepithelialization, angiogenesis, tissue remodeling, and innate host defense, which is in contrast to many other known growth factors such as VEGF. The ability of IL-22 to promote migration of keratinocytes and induce epidermal hyperplasia was first recognized by Boniface and colleagues [46]. They showed that IL-22 induced in vitro keratinocyte migration and induced hyperplasia of keratinocytes in reconstituted human epidermis. In addition, they also noticed that IL-22 down regulated genes involved in keratinocytes differentiation. Sa and colleagues further extended these findings to other IL-20 subfamily members, including IL-19, IL-20 and IL-24, by demonstrating that these cytokines also had similar biologic effects on human epidermal keratinocytes [24]. All these cytokines were able to stimulate the proliferation of human epidermal keratinocytes and induce epidermal hyperplasia. IL-20 family cytokines likely utilize two distinct mechanisms in facilitating reepithelialization. First, these cytokines activate Stat3 which directly mediates the proliferation of epithelial cells [19, 23, 24]. In a recent study, IL-22 was shown to accelerate reepithelialization during wound healing in a streptozotocin-induced diabetic model through the activation of Stat3 [27]. Secondly, IL-20 subfamily cytokines can induce the expression of many growth factors such as PDGF, KGF, and EGF family factors from keratinocytes, fibroblasts or other stromal cells, which might enhance the proliferative activity through an autocrine feedback mechanism [24, 28, 46]. In our study we further confirmed that IL-22 upregulates genes involved in re-epithelialization such as *Areg* (Fig 4H), which is a member of EGF family.

IL-22 can promote tissue remodeling and extracellular matrix formation during wound healing [26]. In IL-22 deficient mice, the dermal wound area, and the expression of fibronectin and certain collagen subtypes were all significantly reduced. IL-22 treatment improves granulation tissue formation and vascularization in the wounds from streptozotocin-induced diabetic mice [27]. Although it is currently unclear whether IL-20 and IL-24 can also act on dermal fibroblasts and induce similar downstream effects as those induced by IL-22, these cytokines all stimulate epidermal keratinocytes to express many proteinases and proteinase inhibitors that can facilitate the tissue remodeling process during wound healing. Consistently, in our study, we also found that IL-22 treatment increased angiogenesis in the wounds of *db/db* mice. Vascular defects in diabetic patients are felt to be of the major reasons that diabetic wounds fail to heal [8, 9]. VEGF has been used to facilitate the wound healing by promoting blood vessel formation in diabetic wound healing models [13, 14]. Although IL-22 did not trigger massive expression of genes involved in angiogenesis as VEGF (Fig 4), we speculate that IL-22 might promote wound healing through indirect mechanisms. IL-22 can induce the expression of VEGF from keratinocytes [24, 27]. In addition, IL-22 is also reported to increase dermal blood vessels when injected into normal human skin grafted onto immune-deficient AGR129 mice [40].

Lastly, and again in contrast to many other growth factors such as EGF, PDGF, and VEGF, IL-20 subfamily cytokines, and particularly IL-22, stimulate innate host defense mechanisms from epithelial cells, including epidermal keratinocytes (Fig 4). In the epithelium of many organs, including intestine and lung, IL-22 is indispensible in controlling the invasion of various pathogens [18, 47, 48]. Previous studies have established that IL-20 subfamily cytokines induce the production of many antimicrobial peptides, including defensins and S100 family peptides, as well as chemokines from keratinocytes [49]. Our microarray study further confirms that IL-22, but not VEGF or PDGF; significantly promote the expression of many antimicrobial genes from the wounds of diabetic mice. Furthermore, IL-22 enhances the expression of chemotaxis related genes, e.g. CXCR2 (Fig 4G) which can promote wound site inflammation via recruiting neutrophils. Consistent with these results, we also demonstrate here that in an infectious wound model in *db/db* mice, IL-22-Fc showed superior efficacy when compared to VEGF and PDGF. As chronic infection is another factor that can hinder wound closure in diabetic patients [9, 45], these results also suggest that IL-22-Fc may provide therapeutic benefit in infected diabetic wounds as well.

In conclusion, we have shown that IL-20 subfamily cytokines can promote several important processes in wound healing, which may contribute to their effectiveness in accelerating the closure of wounds in diabetic *db/db* mice. DFU is a common complication of diabetes mellitus and is a major unmet medical need with no truly effective treatment options [7, 45]. A significant number of patients with DFU ultimately require amputation which becomes the major source of morbidity and the leading cause of hospitalization in diabetes. Although a number of tissue growth factors have been clinically tested in DFU patients, only PDGF (Regranex) has been approved, and its efficacy in DFU has been marginal [5, 50]. Given the unique and broad functions of IL-20 subfamily cytokines in promoting all aspects of wound healing, these cytokines may be able to stimulate wound closure in DFU patients. In support of this premise, in preclinical diabetic models, IL-22-Fc, IL-20-Fc and IL-24-Fc fusion protein were all able to significantly accelerate wound healing, suggesting that these cytokines and fusion proteins may indeed be potential clinical candidates for the treatment of DFU.

## Supporting Information

S1 Fig Representative wound images of different time points of Fig 3G. Clean wounds on *db/db* mice were topically treated with control or IL-22-Fc. Wound images were taken on Day 0, Day 8, Day 14 and Day 18 post treatments. Wound gap measurements were labeled underneath the pictures.
(EPS)
